# Supplementary material for: Double-Edged Sword of Vitamin D3 Effects on Primary Neuronal Cultures in Hypoxic States
Source: Int J Mol Sci. 2021 May 21;22(11):5417. doi: 10.3390/ijms22115417 (PMC8196622; doi:10.3390/ijms22115417)
Supplement: Supplementary file 1 [file ijms-22-05417-s001.zip › Table S1.pdf]

Table S1. Analysis of the number of necrotic and apoptotic cells in primary neuronal cultures day after vitamin D3 application

Group	Number of propidium iodide+ cells, % (necrotic cells)	Number of AnnexinV+ cells, % (apoptotic cells)	Necrotic / apoptotic cells ratio
Sham	4.65±0.63	0.58±0.21	8.04±0.62
Solvent	5.62±0.61	0.81±0.52	6.93±0.53
Vitamin D3 0.01 μM	4.08±0.72	0.89±0.39	4.46±0.39*
Vitamin D3 0.1 μM	5.01±0.48	0.46±0.12	10.90±0.84*
Vitamin D3 1 μM	6.2±0.87	0.54±0.17	11.55±1.02*

* - versus "Sham", $p < 0.05$, one-way ANOVA and Tukey post hoc test
