# Supplementary material for: Double-Edged Sword of Vitamin D3 Effects on Primary Neuronal Cultures in Hypoxic States
Source: Int J Mol Sci. 2021 May 21;22(11):5417. doi: 10.3390/ijms22115417 (PMC8196622; doi:10.3390/ijms22115417)
Supplement: Supplementary file 1 [file ijms-22-05417-s001.zip › Table S2.pdf]

Table S2. Analysis of the number of necrotic and apoptotic cells in primary neuronal cultures day after hypoxia modeling

Group	Number of propidium iodide+ cells, % (necrotic cells)	Number of AnnexinV+ cells, % (apoptotic cells)	Necrotic / apoptotic cells ratio
Sham	4,65±0,63	0,58±0,11	8,04±0,12
Hypoxia	17,31±1,21*	1,95±0,23*	14,48±1,29*
Hypoxia+solvent	17,79±1,98*	1,14±0,21*	15,6±1,11*
Hypoxia+Vitamin D3 0.01 μM	4,98±0,54#	0,67±0,12#	7,38±0,69#
Hypoxia+Vitamin D3 0.1 μM	5,64±0,78#	0,44±0,10#	12,9±1,02
Vitamin D3 1 μM	71,78±2,27*#	6,11±1,01*#	11,74±0,98

* - versus "Sham", # - versus "Hypoxia", $p < 0.05$, one-way ANOVA and Tukey post hoc test
