# Supplementary figures and images for: Double-Edged Sword of Vitamin D3 Effects on Primary Neuronal Cultures in Hypoxic States
Source: Int J Mol Sci. 2021 May 21;22(11):5417. doi: 10.3390/ijms22115417 (PMC8196622; doi:10.3390/ijms22115417)

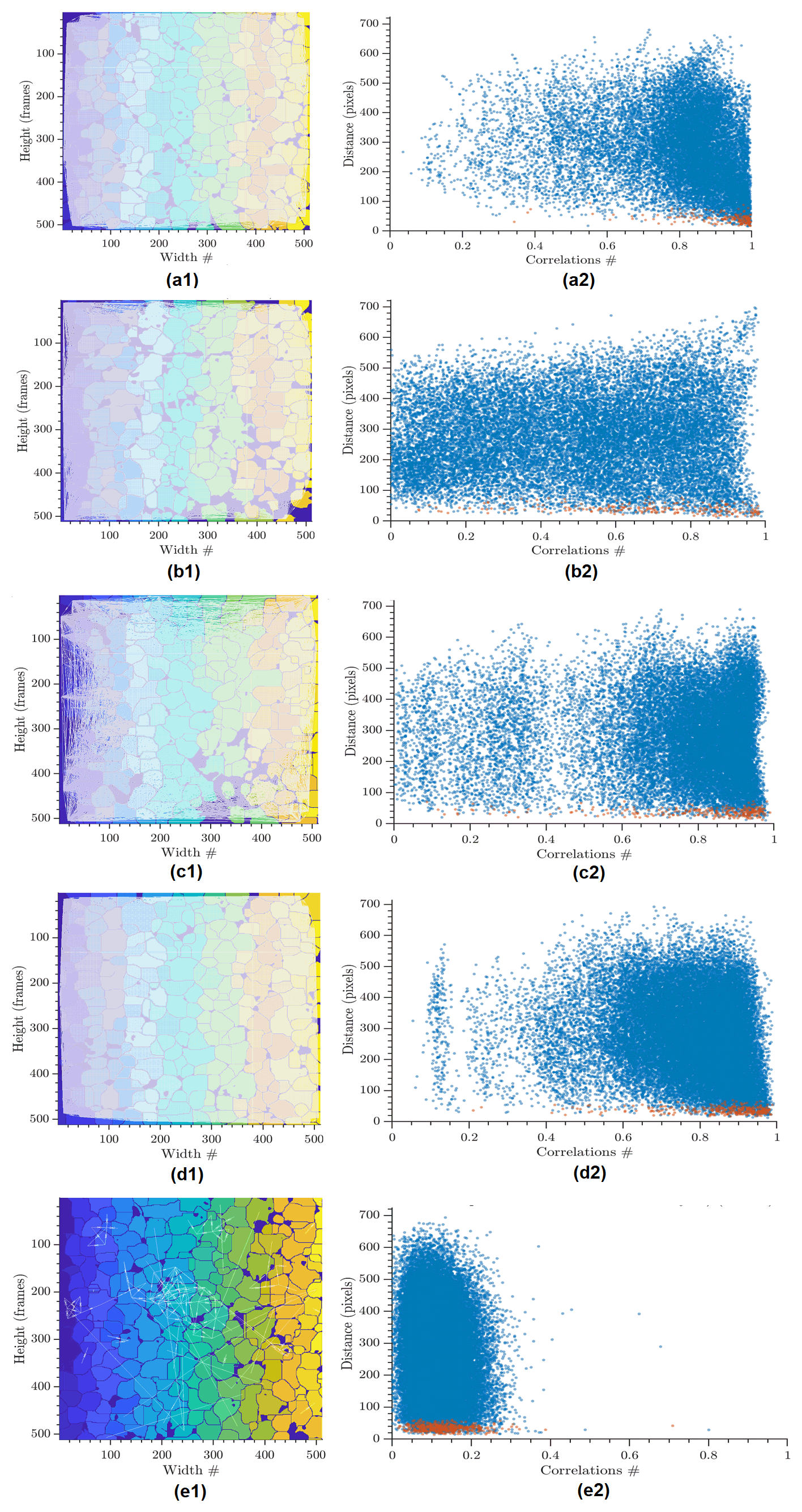

Supplement: Supplementary file 1 [file ijms-22-05417-s001.zip › Figure_S1.tif]

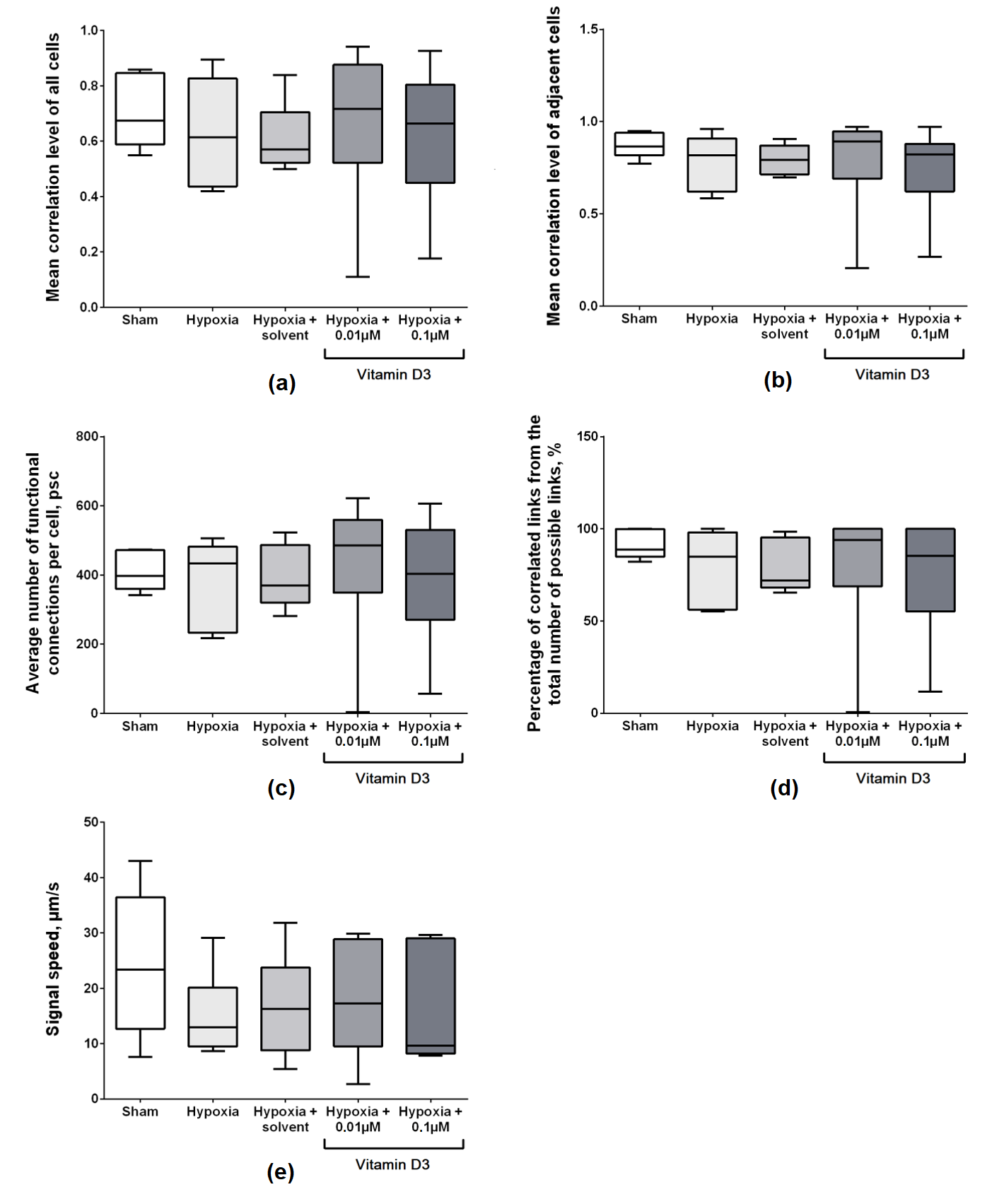

Supplement: Supplementary file 1 [file ijms-22-05417-s001.zip › Figure_S2_revised.tif]

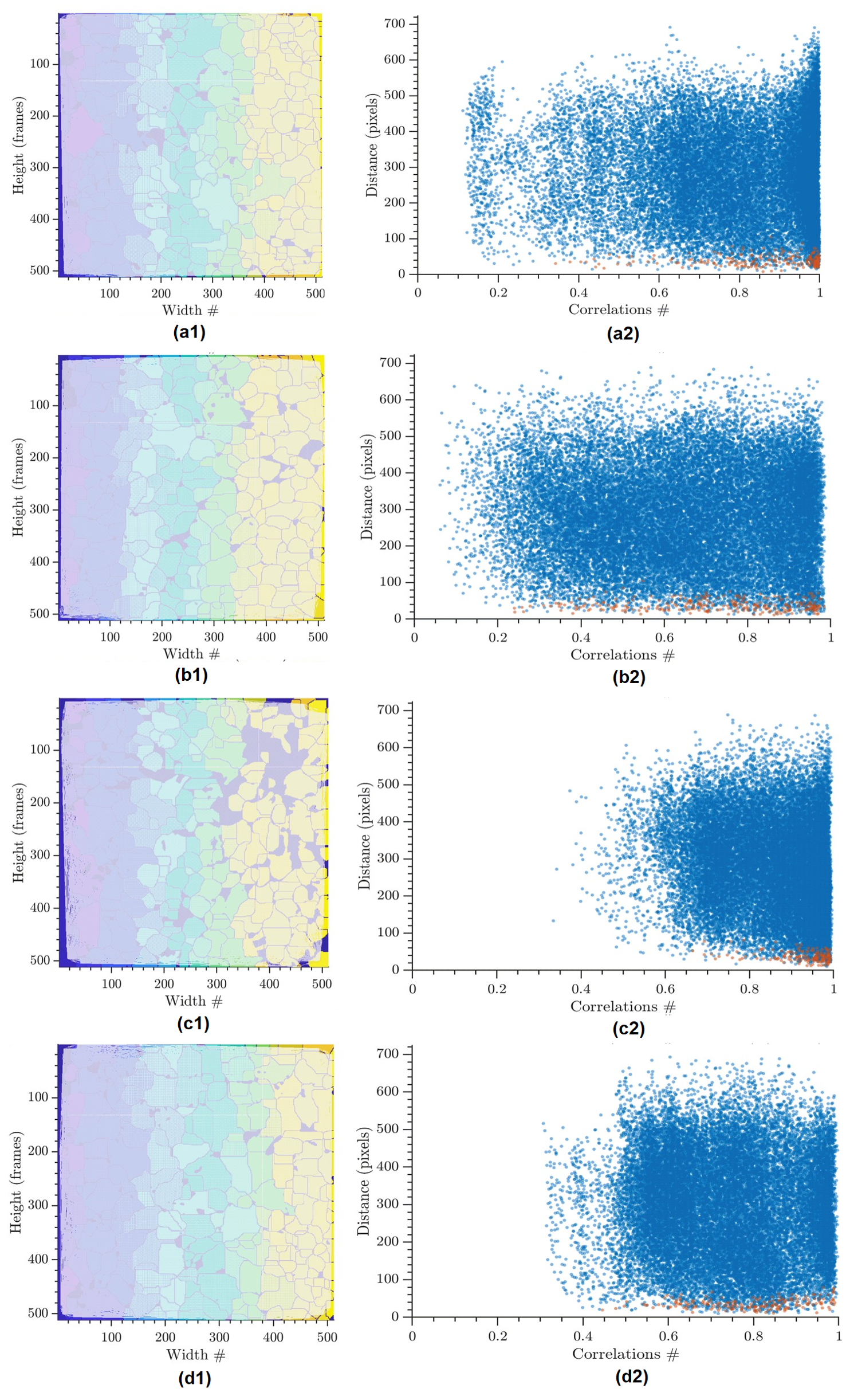

Supplement: Supplementary file 1 [file ijms-22-05417-s001.zip › Figure_S3.tif]

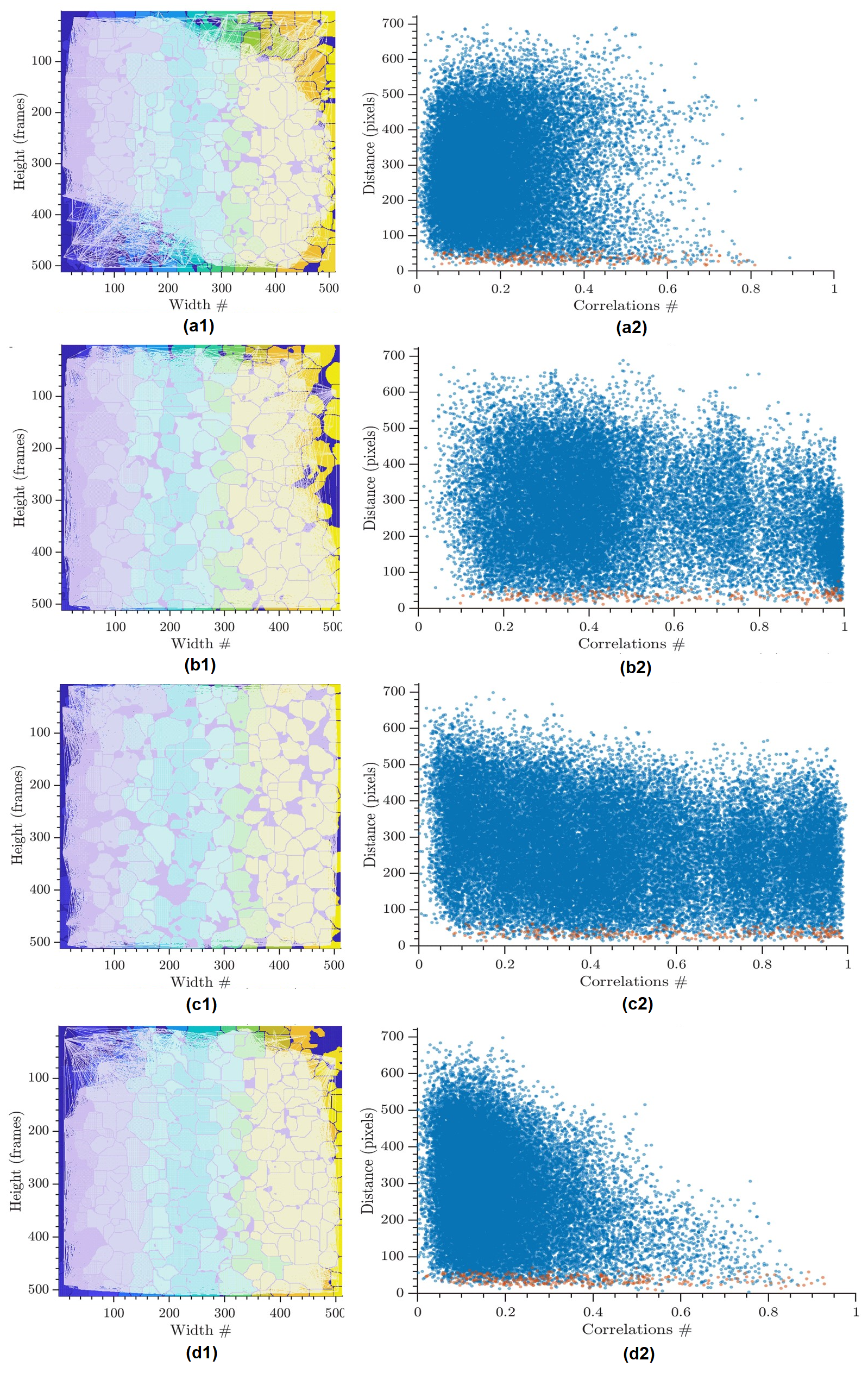

Supplement: Supplementary file 1 [file ijms-22-05417-s001.zip › Figure_S4.tif]

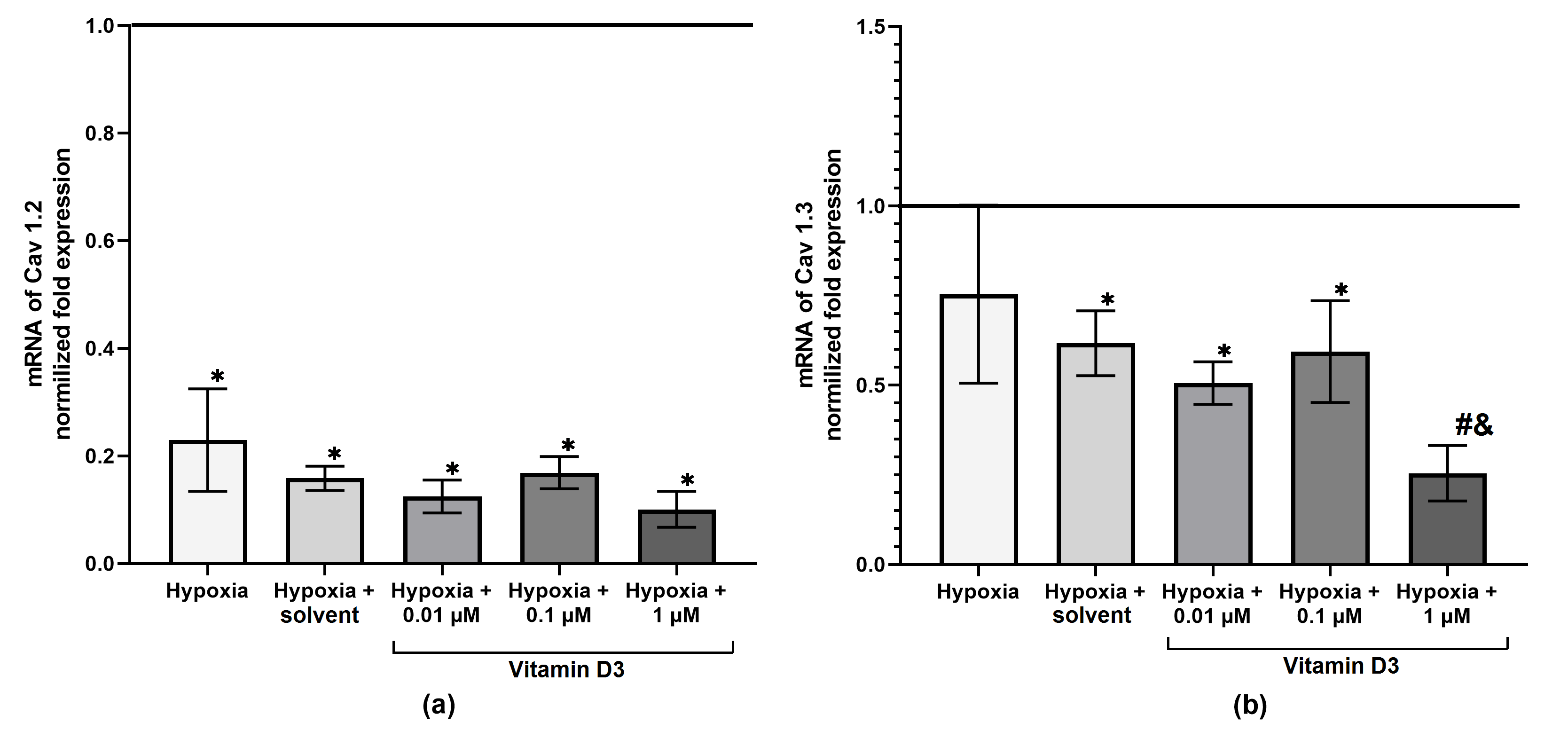

Supplement: Supplementary file 1 [file ijms-22-05417-s001.zip › Figure_S5_revised.tif]
